# Concept for Efficient Light Harvesting in Perovskite Materials via Solar Harvester with Multi-Functional Folded Electrode

**DOI:** 10.3390/nano11123362

**Published:** 2021-12-11

**Authors:** Mao-Qugn Wei, Yu-Sheng Lai, Po-Hsien Tseng, Mei-Yi Li, Cheng-Ming Huang, Fu-Hsiang Ko

**Affiliations:** 1 Department of Materials Science and Engineering, National Chiao Tung University, Hsinchu 300093, Taiwan; barry720919@gmail.com (M.-Q.W.); a19890302@gmail.com (P.-H.T.); 2 Taiwan Semiconductor Research Institute, National Applied Research Laboratories, Hsinchu 300091, Taiwan; meiyi@narlabs.org.tw (M.-Y.L.); pfhaung@narlabs.org.tw (C.-M.H.)

**Keywords:** broadband light harvesting, three-dimensional Schottky junction, perovskite solar cell, photoresponsivity

## Abstract

Conventional electrodes in typical photodetectors only conduct electrical signals and introduce high optical reflection, impacting the optical-to-electrical conversion efficiency. The created surface solar harvester with a multi-functional folded electrode (MFFE) realizes both a three-dimensional Schottky junction with a larger light detecting area as well as low optical reflection from 300 nm (ultra-violet light) to 1100 nm (near-infrared light) broadly without an additional anti-reflection layer. The MFFE needs silicon etching following the lithography process. The metal silver was deposited over structured silicon, completing the whole device simply. According to the experimental results, the width ratio of the bottom side to the top side in MFFE was 15.75, and it showed an optical reflection of 5–7% within the major solar spectrum of AM1.5G by the gradient refractive index effect and the multi-scattering phenomenon simultaneously. While the perovskite materials were deposited over the MFFE structure of the solar harvester, the three-dimensional electrode with lower optical reflection benefitted the perovskite solar cell with a larger detecting area and an additional anti-reflection function to absorb solar energy more efficiently. In this concept, because of the thin stacked film in the perovskite solar cell, the solar energy could be harvested by the prepared Schottky junction of the solar harvester again, except for the optical absorption of the perovskite materials. Moreover, the perovskite materials deposited over the MFFE structure could not absorb near-infrared (NIR) energies to become transparent. The NIR light could be harvested by the light detecting junction of the solar harvester to generate effective photocurrent output additionally for extending the detection capability of perovskite solar cell further. In this work, the concept of integration of a conventional perovskite solar cell with a silicon-based solar harvester having an MFFE structure was proposed and is expected to harvest broadband light energies under low optical reflection and enhance the solar energy conversion efficiency.

## 1. Introduction

Electricity generation has been provided mainly by fossil fuels (like natural gas, oil, nuclear energy, and coal), leading to the global warming issues. Solar energy is a renewable, clean, and environmental fuel to be a suitable candidate for generating green energy. Because the optical behaviors include the reflection, transmission, and absorption as the incident radiation illuminates the light energy harvester, the incident light energy harvested must have as high optical absorption as possible under the conditions of low optical reflection over the surface of the energy harvester for better optical energy-to-electricity conversion efficiency. Furthermore, the effective separation of photo-generation carriers in the light detecting region provides the photocurrent or photovoltage output usefully. It is well-known that nanostructure engineering can increase incidence light harvesting to improve conversion efficiency [1,2,3,4,5,6,7,8,9,10,11,12,13]. In 2013, Chong Liu et al. [4] combined TiO_2_ nanowire and p-type Si for solar to hydrogen (STH) applications. In their study, TiO_2_ nanowire and p-type Si had different energy band gaps to achieve the broadband optical operating range. Furthermore, although the TiO_2_ nanowire structure can increase incidence light harvesting to improve the power conversion efficiency, the efficiency of 0.1% is not high enough for commercial photovoltaic application. Moreover, the previous reports used metal nanoparticles [8,9] or core–shell nanostructures [5,6,10] with localized surface plasmon resonance (LSPR) effects through changing the dimension of nanoparticles (gold or silver) to obtain higher optical absorption in specific light wavelengths. These optical behaviors are strongly dependent on the shape and the size of nanoparticles conducting the narrower optical absorption regime in the solar spectrum to limit the optical-to-electrical conversion capability. The gold nanorod structure with surface plasmonic effect was provided by Mubeen et al. to achieve an STH efficiency of 0.1%. Although Mubeen et al. created the Schottky barrier at the gold nanorod/TiO_2_ interface to separate electron–hole pairs, the higher barrier still impacted the conversion efficiency [12]. Consequently, integrating LSPR with nanostructures or nanoparticles operates in the narrower solar spectrum without obvious broadband light harvesting, resulting in poorer power conversion efficiency.

In recent reports [14,15,16,17,18,19,20,21,22,23,24,25,26,27,28,29], solar energy harvest can be perovskite solar cells for higher conversion efficiency. The perovskite solar cell always absorbs the light wavelength from 400 nm to 800 nm because of the energy band gap of materials for preparing the perovskite-based solar cell. Many researchers improved the efficiency by adjusting the materials directly to conduct two kinds of perovskite solar cells, namely two terminal (2T; single junction) [15,16,17,18,19,20,21,22,23,24] and four terminal (4T, stacked cells) [25,26,27,28,29]. In 1959, H. Kallmann (1959) [14] demonstrated the first organic solar cell (anthracene single crystals), which has an efficiency of 2 × 10^−6^%. The perovskite structure with metal-halide (ABX_3_) can enhance the performance, introducing the reliability issue because of the non-stable material. Several works made the effort of material stability through energy band gap adjustment of ABX_3_. For example, Michael M. Lee. et al. [20,21] proposed a perovskite solar cell with wide band gap energy of 1.55 eV by CH_3_NH_3_PbI_2_Cl to achieve an efficiency of 10.9% (400–800 nm). Eric. T. Hoke et al. [22] and David P. McMeekin et al. [23] also presented the higher energy band gap, from 1.85 eV to 1.68 eV, and that of 1.74 eV, respectively, to bring the non-stable issue into the design. For overcoming the disadvantages of structure stability, photo-carriers transmission, and the wide band gap energy (the narrower optical regime) of materials in 2T perovskite solar cells, the four-terminal (4T) perovskite solar cell was provided. Colin D. Bailie et al. [24] provided the 4T perovskite solar cell composed of silver nanowire with Spiro-OMeTAD (for the hole-transporting layer), mesoporous titanium dioxide (for the electron-transporting layer), and the stacked tandem configuration onto copper indium gallium diselenide (CIGS). UV light illuminates TiO_2_ because oxygen can be generated to impact the band gap of the perovskite material, bringing the limited conversion efficiency of 13.7%. Although the ETL composed of SnO_2_ [25] or PCBM/PEIE [26] (without the oxygen’s influence under UV regime) could achieve efficiencies of 18.4% and 19.2%, respectively, broadband optical energy harvesting is still limited by the energy band gap of the material itself. Furthermore, once the broadband light conversion can be implemented, the conversion efficiency should be improved further. For this purpose, combining a hetero-junction structure or CIGS with a perovskite solar cell provides the possibility to extend the optical absorption capability. These methodologies always conduct complex fabrication in whole device integration.

In this work, a surface solar energy harvester with a multi-functional folded electrode (MFFE) was prepared for three-dimensional light harvesting (Schottky junction) with broadband optical absorption (300–1100 nm) and effective photocurrent conduction simultaneously at the same device structure. The solar harvester could be fabricated through the conventional semiconductor process without altering any process step. After completing the whole harvester with the MFFE structure, optical anti-reflection and light harvesting were realized in the single MFFE structure simultaneously. Typically, the metal electrode with high optical reflection behavior impacts optical-to-electrical conversion capability. The higher optical reflection behavior is usually from the intense change in the optical property of n (the refractive index) along the incident light transition path. Moreover, the anti-reflection structure over flat metal film is too complex to provide the broadband low optical reflection. Besides, the trade-off between the area of the metal electrode and the light detecting region is another change, because the smaller metal electrode brings the longest diffusion distance of photo-generated carriers with a higher recombination possibility. The surface solar harvester is created by integrating the conventional Schottky junction with the structured silicon surface in a specific three-dimensional periodical optical structure to form the MFFE structure.

The MFFE structure provides the gradient refractive index for lowering the intense refractive index change in the interface between air and metal to achieve the low optical reflection within the solar spectrum (AM1.5G) as broadly as possible. For easy incident light harvesting, the thin metal is deposited over the MFFE structure, forming the Schottky junction to harvest the incident light. It is worth mentioning that the correlation between incident optical intensity and light penetration depth is exponential decay, indicating that the light detecting junction must be located near the Si surface for the better light harvesting capability. Because of the designed Schottky junction in the MFFE structure just below the metal electrode, experimental results also indicate the high photoresponsivity and low optical reflection as the incident light wavelength from 300 nm to 1100 nm, compared with the contrast device without any optical structure. At the same time, theoretical analysis is also provided to confirm the MFFE structure with an obvious gradient refractive index phenomenon. The significant breakthrough can be expected to break the trade-off between the area of the metal electrode and the light detection region to provide excellent solar harvesting and carrier collection within the same MFFE structure. Without an extra anti-reflection structure, the silicon width ratio of 15.75 in the MFFE structure reveals the lowest optical reflection of 3–5% from the ultra-violet to the near-infrared regime broadly. Furthermore, this work also realizes 2.5 times and 5 times the external quantum efficiency (EQE) and the responsivity of the contrast device without an optical structure, respectively. As the perovskite solar cell is deposited over the three-dimensional MFFE optical structure, the former possesses more detecting area and additional anti-reflection function in the visible regime to enhance the conversion efficiency of the perovskite solar cell further. More, it is due to the thin perovskite materials that the visible light energy can be absorbed by the solar harvester and the perovskite solar cell at the same time. The responsivity of 0.5 A/W in the created harvester peaks around 900 nm, benefiting the NIR light harvesting even though the perovskite cell cannot absorb NIR light. Consequently, the concept of integration of the perovskite solar cell with the surface solar harvester profits broadband energy harvesting efficiently.

## 2. Materials, Fabrication, and Methods

### 2.1. Device Fabrication

The mainstream complementary metal-oxide-semiconductor (CMOS) technology was used to fabricate the solar harvester without altering any standard processes. Fabrication began with the pattern formation of the three-dimensional structure in the surface of silicon through electron-beam lithography (EBL, Leica Weprint 200 e-beam, Leica Camera AG Corp., Wetzlar, Germany). The dry etching system (TCP-9400, Lam Research TCP 9400 Poly Etcher, Lam Research Corp., Fremont, CA, USA) provided the silicon removal process according to the lithography pattern to achieve the trench depth of 1.2–1.3 μm. After stripping off the photo resister from the surface of the silicon, the three-dimensional structures were prepared with the diameter and the period of 0.4 μm and 0.8 μm, respectively. Then, for reducing the traps (dangling bound) induced from the silicon etching process, the silicon dioxide of 20 nm was formed and removed by 49% of the hydrofluoric acid (HF and DI water with a volume ration of 1:50) at 25 °C immediately to repair the damaged silicon surface. Using sputter and physical vapor deposition (FSE-Cluster-PVD, F.S.E Corp., New Taipei City, Taiwan), the silver film (topside electrode) and TiN film (backside electrode) with thicknesses of 20 nm and 50 nm, respectively, were obtained. As the silver deposited over these optical structures, the structured metal was fabricated in the same process simultaneously to achieve process simplification in our device. Due to the differences between the work function of metal electrode and the fermi-level of n-type silicon, the Schottky junction and the ohmic junction could be provided in the interfaces between silver and n-type silicon and that among n-type silicon and TiN, respectively. Furthermore, the created three-dimensional Schottky junction in the solar harvester achieved a larger light harvesting area in the fixed device size and the photoelectron injection due to the band diagram of the Schottky junction. Obviously, the fabrication of the proposed device with MFFE structure was as straightforward as fabricating the conventional Schottky diode. Because of the diameter and the period of 0.4 μm and 0.8 μm in the designed optical structures, respectively, the pattern definition process could use i-line lithography for lower fabrication cost and high throughput instead of the expensive electron-beam lithography process in the future. Figure S1 describes the brief flow to illustrate the design concept feasibility of integrating the solar harvester with the perovskite solar cell in the future. Indium tin oxide (ITO) is usually the bottom electrode of the conventional perovskite solar cell. The perovskite materials can be coated following the ITO being deposited over the three-dimensional silver film in the MEEF structure of the solar harvester. The interface between the silver (top electrode of the solar harvester) and ITO electrode in the perovskite solar cell should be a good ohmic contact without any issues of fabrication. It also illustrates the good electrical connection to confirm that the perovskite solar cell and the solar harvester are in the series connection electrically shown in Figure S1. 

### 2.2. Metrology, Optical Simulation, and Device Characterization

The prepared optical structures were imaged using transmission electron microscopy (TEM, JEM-2010F, JEOL Corp., Tokyo, Japan) for confirming the device structure, especially the width ratio of the bottom silicon to the top one in the MFFE structure. The three-dimensional finite-difference time-domain (3D-FDTD, Ansys/LUMERICAL Corp., Vancouver, Canada) method was employed to simulate the optical behaviors (optical reflection), the distribution of the electrical field in the proposed device, and those in the contrast device lacking the optical structure. In the device verification, an optical spectrometer (Hitachi, U4100, Hitach Corp., Tokyo, Japan) was used to investigate the optical reflection of all prepared samples. Consisting of the monochromatic having the light source of the Xe lamp, the optical filter (300–1100 nm) and the source-measurement-unit (Keithley 2400, Keithley Corp., Solon, OH, USA) provided the optical-to-electrical conversion efficiency data such as photoresponsivity and the current–voltage characteristics in this work.

## 3. Results and Discussion

### 3.1. Device Design

**Figure 1:**
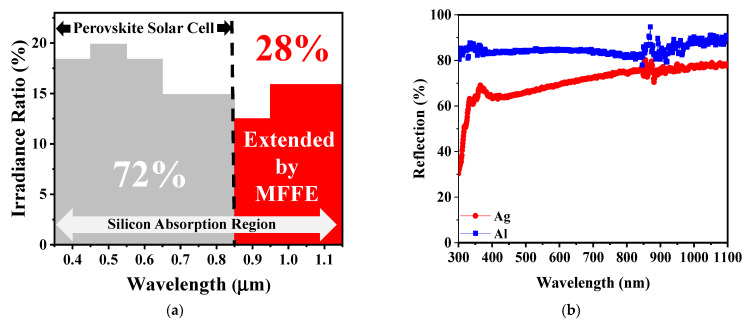
(**a**) The irradiation ratio distribution within AM1.5G. The perovskite solar cell absorbs only 72% of the irradiation spectrum. Combining the multi-functional folded electrode (MFFE) with the perovskite solar cell benefits the harvest of additional near-infrared light energy for enhancing the conversion efficiency further; (**b**) the optical reflection of metal silver (Ag) and that of aluminum (Al). Significantly, the high optical reflection can be observed without optical structure, and silver has the inter-band transition phenomenon (low optical reflection) in the ultra-violet regime.

Figure 1a illustrates the light energy distribution from 400 nm to 1100 nm within the solar spectrum of AM1.5G. In this regime, the silicon-based energy harvester could absorb all the incident energies because of the silicon energy band gap of 1.12 eV only. In contrast to the silicon material, the perovskite solar cell with the larger energy band gap always absorbed the light wavelength from 400 nm to 800 nm. Within the solar spectrum of AM1.5G in Figure 1a, only 72% the incident solar energy could be harvested by the perovskite solar cell to contribute to the optical-to-electrical conversion. The energy band gap of material utilized in the perovskite solar cell was too large to absorb the near-infrared (NIR) energies, introducing the waste of incident energies (28% the intensity spectrum in AM1.5G). For harvesting the additional NIR energies, the conventional perovskite solar cell could be integrated with the silicon-based energy harvester. While the perovskite solar cell was stacked over the MFFE structure of the solar harvester, the NIR energies could still reach the light detecting area of the Schottky junction of the solar harvester easily. In this study, the concept of integration of a perovskite solar cell with a silicon-based optical energy harvester may be the candidate for not only the optical energy absorption as broadly as possible but also the enhancement of optical-to-electrical conversion capability further. In device fabrication, the perovskite solar cell can be fabricated through multi-thin film deposition with total film thickness of a few hundred nm. These film depositions can be easy to reproduce over the MFFE structure for the purpose of perovskite solar cells stacked over the solar harvester. For electrical consideration, the bottom electrode in the perovskite solar cell and the top metal of the MFFE are ITO and silver, respectively. It was clearly confirmed that there was a good electrical connection, as the perovskite solar cell and the solar harvester were connected in series electrically. In optical energy harvesting, because of the three-dimensional MFFE structure in the desired solar harvester, the deposited perovskite materials can achieve a larger dimension for light harvesting compared with that of a conventional perovskite solar cell. Furthermore, the perovskite solar cell only absorbs the light energies from 400 nm to 800 nm. The lower optical reflection in the visible spectrum is crucial to the perovskite solar cell. The suggested solar harvester with the MFFE structure realizes the broadband low optical reflection, benefiting the visible light absorption of perovskite materials as long as the perovskite solar cell is stacked over the solar harvester. Because of the thin stacked perovskite materials over the MFFE structure of the solar harvester, the incident light energies can be harvested by the solar harvester again to provide more photocurrent output relative to the conventional perovskite solar cell. Moreover, the near-infrared energies can pass through the perovskite solar cell to arrive at the silicon-based solar harvester for generating electron–hole pairs additionally in the interface between n-type silicon and silver (MFFE region). Therefore, comparing the single perovskite solar cell with that stacked over the silicon-based solar harvester, the latter can be expected to realize light harvesting broadly for providing higher optical-to-electrical conversion efficiency within solar spectrum of AM1.5G potentially.

**Figure 2:**
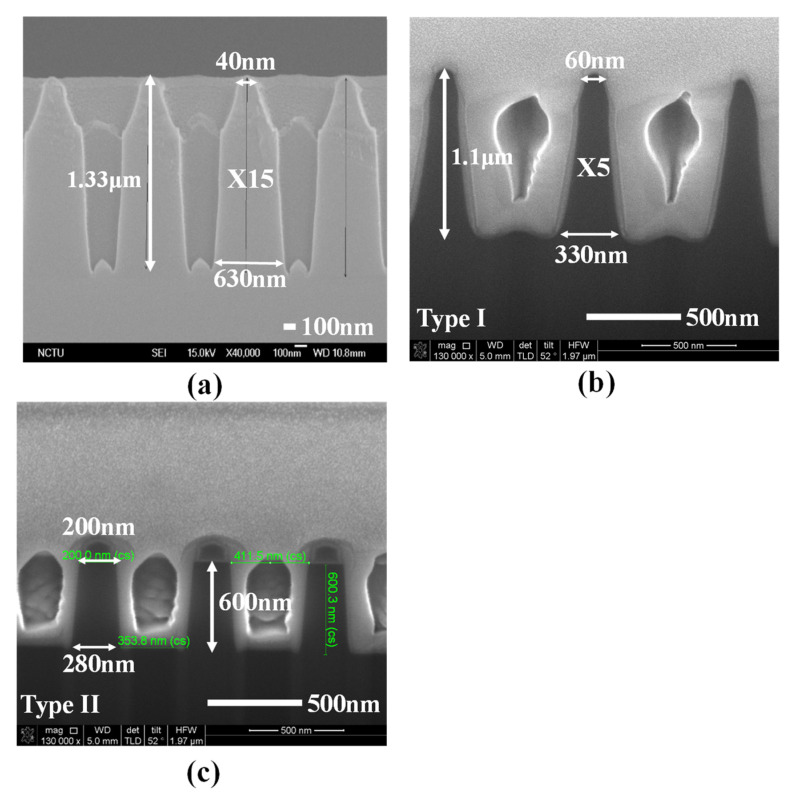
The cross-sectional view of prepared optical structures. The multi-functional folded electrode (MFFE), Type I, and Type II are shown in (**a**–**c**), respectively (“X15” and “X5” marked in (**a**,**b**), respectively, are the width ratio of the bottom silicon to the top one, indicating the different tilt in the prepared structures).

Generally, the conventional metal electrode of the silicon-based optical energy harvester with high optical reflection naturally impacts the optical absorption. The anti-reflection structure over the metal electrode is difficult to conduct the complex device fabrication. Figure 1b displays the optical reflection behavior of metal aluminum (Al) and that of metal silver (Ag) in the ultra-violet (UV, 300–400 nm), the visible (400–700 nm), and the NIR (700–1100 nm) regime. Significantly, the optical reflection of Al without any anti-reflection structures is as high as 80% at least. Under the same verification condition, in the case of Ag, it shows the lower optical reflection in the UV regime and in the visible regime compared with metal Al, which is due to the difference of optical properties (n and k) in Al and Ag and the inter-band transition phenomenon of silver in the UV regime additionally. We believe the MFFE with silver will achieve high UV light harvesting capability relative to that with metal of Al. The Schottky junction can be created in the interface between silver metal and n-type silicon, having the depletion region in silicon to harvest light energy. Significantly, the light harvesting regime is near the silicon surface, benefiting absorption of the incident light power. Typically, the incident energy shows exponential decay accompanying the increase of light penetration depth into the silicon. The shorter the light penetration depth in silicon is, the higher the incident intensity can be obtained for harvesting and converting by the surface solar energy harvester. Consequently, the Schottky junction could be the candidate for the surface optical harvester, profiting to harvest light with the advantages of easy fabrication and a large junction area in the three-dimensional MFFE structure for harvesting incident light energy simultaneously. The prepared devices in this study are illustrated in Figure 2a–c, which are the cross-sectional SEM images to illustrate the different optical structures, namely the multi-functional folded electrode (MFFE), Type I, and Type II, respectively. The formation of these structures in Figure 2 is silicon etching following the lithography process for transferring the optical structure patterns. Then, the metal silver was sputtered to complete the whole device. This process prevents the manufacture of the optical structures over the surface of metal electrode directly and benefits the large (three-dimensional) Schottky junction area formation with the folded metal structure simply. All these device design concepts can be easily achieved through one lithography process, one silicon etching, and metal deposition with a metal thickness of around 15 nm. 

### 3.2. Theoretical Analysis of Effective Refractive Index

**Figure 3:**
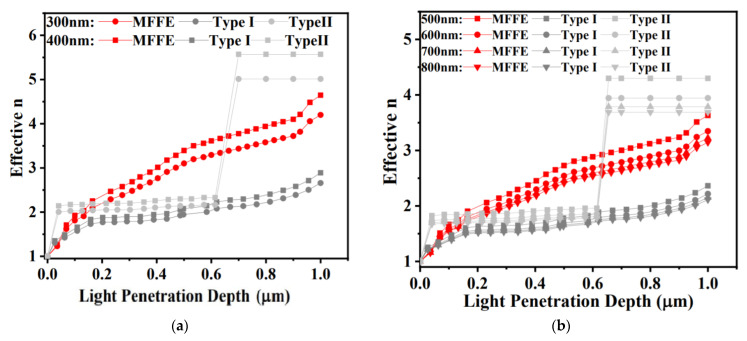

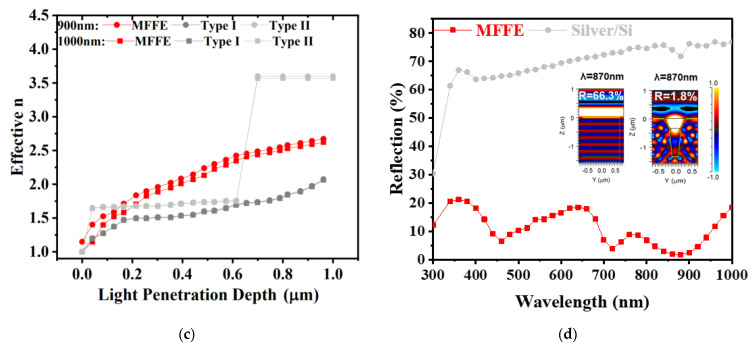
The calculated correlation between effective refractive index (n) and light penetration depth in (**a**) ultra-violet light; (**b**) visible light; and (**c**) near-infrared light. These results illustrate that MFFE provides the linear gradient refractive index compared with the others in this work; (**d**) the simulated optical reflection by three-dimensional finite-differential time-domain (FDTD) software.

In Figure 1b, the metal without any optical structure shows the high optical reflection phenomenon. Herein, the theoretical analysis will be provided to confirm the suggested MFFE with lower optical reflection within the solar spectrum of AM1.5G. The low optical reflection can be realized as long as the change of refractive index is insignificant along the light traveling path. For example, the refractive index of air is quite different from a metal surface conducting a significant optical reflection. The results of theoretical analysis are illustrated in Figure 3 for confirming the existence of a gradient refractive effect in the prepared structures (Figure 2) to realize the lower and broader optical reflection behavior in this work. The verifying results of light wavelengths are UV light, visible light, and NIR light, as displayed in Figure 3a–c, respectively. The analysis calculated the effective optical property n (the refractive index) contributed by the n of silicon and that of air with the variant ratio of silicon to air accompanied with the different incident light penetration depth. In Figure 3a, the approximate linear change in effective refractive index n indicates the optical structure, MFFE, with the gradient refractive index effect that is beneficial to optical reflection reduction. Generally, high optical reflection occurs in the interface, having a dramatic change of refractive index, such as pure metal in air. While the silicon is etched like a tapered shape in the MFFE with the period and the top silicon width of 0.8 μm and 0.04 μm, respectively, this structure provides the effective refractive index produced by the silicon and air under some light penetration depth and multi-scattering phenomenon simultaneously. In the same way, under deeper light penetration depth, it can provide another effective refractive index to form the gradient refractive index effect, lowering the significant difference in refractive index between silicon and air. For this purpose, the MFFE has the inclined plane in Figure 2a to provide the gradient ratio of silicon to air for stimulating an effective gradient refractive index effect and the multi-scattering optically. According to the calculated results in Figure 3, the MFFE structure achieved a greater linear change in refractive index as light penetration depth increased compared with the other optical structures, namely Type I and Type II. Significantly, the effective refractive index of Type II presented an obvious sudden change no matter what the incident lights were. As the incident light met the prepared structure of Type II, it was due to the widest top silicon width of 0.2 μm in Figure 2c, introducing the additional optical reflection and optical interference to impact the gradient refractive index effect. Additionally, although two optical structures, MFFE and Type I, had similar etched depths of silicon and the pitch of optical structure arrangement, the appearance diversity of the optical structure introduced completely different optical behavior. Structure “Type I” had a top silicon width of 0.06 μm, which was wider than that of MFFE (~40 nm), and a narrower bottom silicon width of 0.33 μm compared with that of MFFE (~0.63 μm). Investigating the “effective n” of MFFE in Figure 3c still realizes a more linear gradient refractive index change compared with the contrast structure, Type I and Type II. In the longer light wavelength like the NIR regime, the dimension of the optical structure MFFE is fixed, and it will conduct the unexpected optical behavior to impact the gradient refractive index effect slightly. Through the calculated results of effective refractive index in Figure 3a–c, these results imply the proposed MFFE with the capability of broadband optical reflection reduction without an external anti-reflection structure. The simulation results through the finite-difference time-domain (3D-FDTD) software provide evidence for the optical reflection reduction phenomenon in the prepared MFFE structure illustrated in Figure 3d. In Figure 3d, the proposed surface solar energy harvester with MFFE realized the lower optical reflection within the solar spectrum broadly compared with the contrast device without any optical structure. In the inset of Figure 3d, the brightness area in the simulated result indicated the strongest electrical field region (optical absorption). It is clear to investigate the electrical field confined in the suggested MFFE for re-confirming the multi-scattering phenomenon and the incident light harvested by MFFE to demonstrate lower optical reflection.

### 3.3. Verification of Optical Behaviors

**Figure 4:**
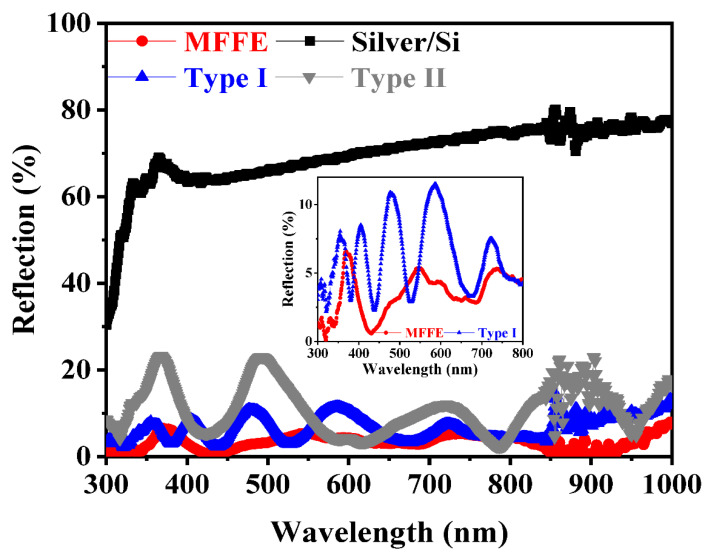
The measured optical reflection through the optical integrating sphere system. The MFFE provides the lowest optical reflection from incident light wavelength of 300 nm to that of 1000 nm. The optical reflection can be as low as 5–7%, illustrated in the inset of Figure 4 to confirm the broadband light harvesting in MFFE without anti-reflection structure over silver additionally.

According to the results shown in Figure 2a and Figure 3a–c, the MFFE device can be expected to provide a significant gradient ratio of silicon to air for stimulating the effective gradient refractive index effect to lower the significance of the optical reflection theoretically. The verified results of optical reflection are displayed in Figure 4. The lack of any anti-reflection structure or gradient refractive index effect in the silver/Si sample introduces the highest optical reflection of 60–70% from UV to NIR regimes. It also points out the low optical-to-electrical conversion capability in the silver/Si sample at the same time. The device with the structure of Type II having the dramatic change in effective refractive index (Figure 3) also conducts obvious optical interference and the optical reflection as high as 20% because of the widest top width of silicon and the shallower (~0.6 μm) depth in the designed structure of Type II compared with that of MFFE (~1.3 μm). According to Figure 2c, the top and bottom silicon width of the Type II structure were 0.2 μm and 0.28 μm, respectively; the width difference between the top silicon and bottom one was too similar in the optical structure to provide the efficient gradient ratio of silicon to air for stimulating the effective gradient refractive index effect. Without any anti-reflection structure, the measured results in the inset of Figure 4 illustrate that the significant low optical reflection (<7%) within broadband incident light wavelengths from 300 nm to 1100 nm can be achieved in the proposed solar harvester with the MFFE structure relative to all the prepared samples. Two optical structures of MFFE and Type I have similar etched depth of silicon and the pitch of optical structure arrangement in the SEM images of Figure 2a,b, respectively. Comparing the reflection of the device with MFFE and that with Type I shown in the inset of Figure 4, the former provided the highest optical reflection of only 7% with insignificant optical interference in the visible to NIR regime successfully. It confirms the results of theoretical analysis about the gradient refractive index (in Figure 3a–c) and the multi-scattering phenomenon (electrical field confined phenomenon in Figure 3d) in the MFFE structure. Moreover, the width ratio of the bottom silicon to the top one was 5.5 (bottom/top: 0.33 μm/0.06 μm) in the Type I structure, introducing a poorer gradient spacing width of the optical structure without stimulating a more linear gradient refractive index effect compared with that of 15.75 (bottom/top: 0.63 μm/0.04 μm) in the MFFE structure. Therefore, devices with Type I structures display higher optical reflection and optical interference, which is clearly shown in the insert of Figure 4. 

### 3.4. Device Characterization

**Figure 5:**
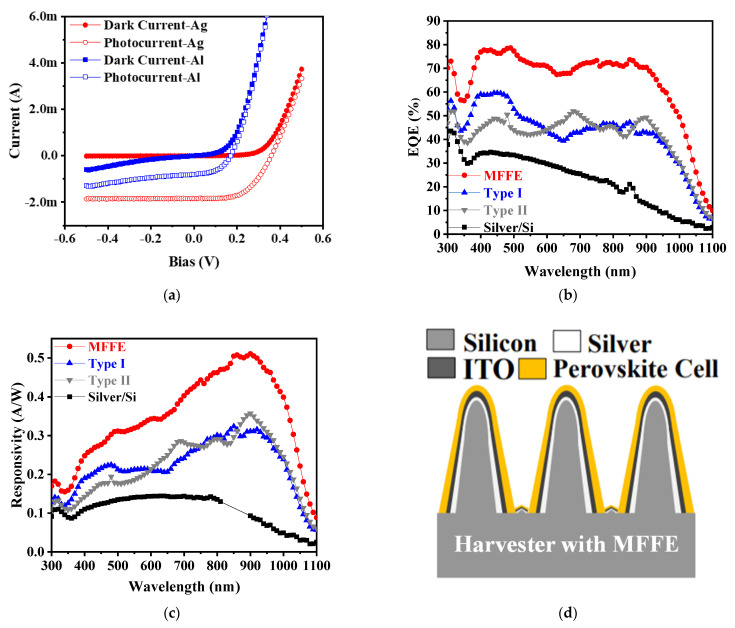
(**a**) Current–voltage (I–V) characteristics of the MFFE device with deposition of different metal materials (Ag and Al); (**b**) the external quantum efficiency (EQE) spectral response; (**c**) the responsivity of the MFFE structure compared to other structures (type I, type II, and silver/si structure); (**d**) concept of integrating a perovskite solar cell with a prepared solar harvester with MFFE.

In Figure 5a, the current–voltage characteristics provide evidence about the existence of the Schottky junction between Al and n-type silicon and that between Ag and n-type silicon in the same MFFE structure. As devices are in dark environments, the cut-in voltage of devices with Al deposited is smaller than that of devices with Ag, indicating the lower Schottky barrier height in the Al case. It may be the obvious Schottky junction barrier lowering effect because of more interface defects in the metallurgical interface. Moreover, more photocurrent output can be provided in the device with the silver-deposited MFFE structure (relative to the device with the Al-deposited MFFE structure) under the same illuminating intensity. According to these results, the metal silver was chosen to form the suggested solar energy harvester in this work. The external quantum efficiency (EQE) and the responsivity are illustrated in Figure 5b,c, respectively, to investigate the optical-to-electrical conversion capabilities in the prepared samples. Clearly, as long as the device with the MFFE structure, and the highest EQE and responsivity can be achieved, confirming the desired gradient refractive index effect, the optical multi-absorption, and the electrical field confined phenomenon in the MFFE structure simultaneously. Moreover, in Figure 5b,c, the sample named “silver/Si” is the contrast device without any optical structure having the highest optical reflection shown in Figure 4. Because of the thinner thickness of silver in this work, the incident light could penetrate through the metal to arrive at the depletion region of the Schottky junction between silver and n-type silicon. Generally, the optical property, extinction coefficient k, is higher in the UV regime than that in the visible regime. As the shorter incident light wavelength illuminates the contrast device, it conducts the significant performance of EQE and that of responsivity, implying the optical absorption behavior is dominated by silicon itself directly. According to the measured results in Figure 5b,c, the device named “silver/Si” is still unable to provide the highest EQE or responsivity like the device with MFFE, confirming that the suggested MFFE structure is crucial to light harvesting capability instead of high UV light absorption in silicon itself. Unlike the operation principle of contrast devices, the MFFE structure provides the extra optical anti-reflection effect to harvest more incident light energy as broadly as possible within the solar spectrum of AM1.5G for enhancing the EQE or responsivity further. According to responsivity data in Figure 5c, the spectrum peaked at around 900 nm due to the MFFE structure with the dimensional arrangement in the diameter (0.4 μm) and period (0.8 μm). Based on the verified result, the design concepts of multi-functional electrodes were demonstrated successfully, including the three-dimensional surface optical energy harvester, broadband optical anti-reflection from UV to NIR regime without additional optical structure over metal silver, and the simple fabrication for preparing the device as straight forwardly as the conventional Schottky diode simultaneously. Moreover, Figure 5d illustrates the design concept feasibility of integration the perovskite solar cell with the suggested solar harvester with the MFFE structure. The process details are shown in Figure S1. Through the verified results of the solar harvester with the MFFE, the created solar harvester had the peak photo-responsivity of around 900 nm within the AM1.5G spectrum in this work. The process of perovskite solar cell deposition over the MFFE structure is easy to be realized by thin film coating. That also provides a greater detecting region in a fixed device size because of the three-dimensional MFFE optical structure. Further, in conventional perovskite solar cells, the optical reflection reduction in the visible light regime is crucial to improve the energy conversion efficiency. The prepared MFFE structure can provide the additional optical anti-reflection functionality to benefit the visible light energies absorbed by the perovskite solar cell, except the optical absorption of the Schottky junction in the solar harvester. Figure 5d illustrates the concept of this study in combining a conventional perovskite solar cell with a silicon-based solar harvester having a three-dimensional MFFE structure. The solar harvester realizes the broadband low optical reflection benefiting the visible light absorption of perovskite materials as the perovskite solar cell is stacked over the solar harvester. Because of the thin perovskite materials, the incident light energies can be harvested by the solar harvester again to provide the enhanced energy conversion efficiency compared with the conventional perovskite solar cell. In the NIR regime, the perovskite solar cell becomes transparent, which is due to the larger band gap of perovskite materials than that in silicon. The NIR energies can pass through the thin perovskite material to be absorbed by the Schottky junction in the solar harvester. In this work, the concept of integration of a conventional perovskite solar cell with a silicon-based solar harvester with an MFFE structure has proposed and expected to harvest broadband light energies under low optical reflection as well as the enhancement of solar energy conversion efficiency. Table 1 illustrates the device performance of the solar harvester relative to that of the reported perovskite solar cell in optical absorption capability (device operation range), external quantum efficiency (EQE), and photocurrent density simultaneously.

## 4. Conclusions

In this work, a surface solar harvester with an MFFE for efficient harvesting of the light energies from 300 nm (UV) to 1100 nm (NIR) was proposed and verified by a three-dimensional Schottky junction, revealing the broadband optical reflection of 5–7% (UV to NIR regime) and photogeneration carrier’s collection/conduction at the same structure to demonstrate the intension of a multi-functional electrode successfully. Without altering any mainstream semiconductor process, the MFFE structure provides the gradient reflective index and multi-scattering phenomenon to reduce the optical reflection broadly for enhancing the optical harvesting capability within the solar spectrum of AM1.5G. According to the experimental results, the ratio of bottom silicon width to that of the top in the MFFE is 15.75, illustrating the lowest optical reflection. This is in agreement with the theoretical analysis of the gradient reflective index effect. It is worth mentioning that the solar harvester with the MFFE lacking an extra anti-reflection structure still achieves 2.5 times and 5 times the EQE and the responsivity of the contrast device without any optical structure, respectively. Moreover, the spectral response of the solar harvester peaks near 900 nm because of the diameter of 0.4 μm and period of 0.8 μm in the MFFE structure. As the perovskite solar cell is deposited over the three-dimensional MFFE optical structure, the former possesses more detecting area and the additional anti-reflection function to enhance the conversion efficiency further. The responsivity of 0.5 A/W in the created harvester peaks around 900 nm, benefiting from the NIR light harvesting in the depletion region of MFFE even though the perovskite cell cannot absorb NIR light. Consequently, the concept of integration of a perovskite solar cell with a surface solar harvester was proposed and is expected to harvest broad light energies (by a perovskite solar cell and a Schottky junction in a solar harvester) under low optical reflection (by an MEEF structure) for enhancing solar energy conversion efficiency further.

## Figures and Tables

**Table 1:** Comparisons of device performances.

	Science [23]	Energy Environ. Sci. [25]	Energy Environ. Sci. [29]	This Work
Optical Absorption	400–700 nm	400–700 nm	400–700 nm	300–1100 nm
Light Harvest Regime	Multi-junction	Multi-junction	Multi-junction	Single-junction
EQE (%) (400–700 nm)	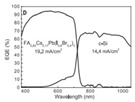 70% @ 400 nm 90% @ 500–700 nm	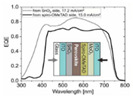 ~5% @ 400 nm 70% @ 500–700nm	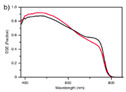 80% @ 400 nm 70% @ 600 nm 60% @ 700 nm	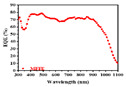 70% @ 300 nm 80% @ 400 nm 70% @ 500–700 nm
EQE (%) (800–1000 nm)	N/A	N/A	N/A	70% @ 800–900 nm 50% @ 1000 nm 10% @ 1100 nm
Current Density (mA/cm^2^)	19.2	17.2	17.5	2

## Data Availability

This study did not report any data.

## Supplementary Materials

The following are available online at https://www.mdpi.com/article/10.3390/nano11123362/s1, Figure S1: The brief flow for illustrating the design concept in this work about integrating solar harvester with the perovskite solar cell.
