# Supplementary material for: Concept for Efficient Light Harvesting in Perovskite Materials via Solar Harvester with Multi-Functional Folded Electrode
Source: Nanomaterials (Basel). 2021 Dec 11;11(12):3362. doi: 10.3390/nano11123362 (PMC8708830; doi:10.3390/nano11123362)
Supplement: Supplementary file 1 [file nanomaterials-11-03362-s001.zip › nanomaterials-1425316-SI.pdf]

nanomaterials

Supporting Information

Concept for Efficient Light Harvesting in Perovskite Materials via Solar Harvester with Multi-Functional Folded Electrode

Mao-Qugn Wei [1], Yu-Sheng Lai [2,*], Po-Hsien Tseng [1], Mei-Yi Li [2], Cheng-Ming Huang [2] and Fu-Hsiang Ko [1,*]

[1] Department of Materials Science and Engineering, National Chiao Tung University, Hsinchu 300093, Taiwan; barry720919@gmail.com (M.-Q.W.); a19890302@gmail.com (P.-H.T.)
[2] Taiwan Semiconductor Research Institute, National Applied Research Laboratories, Hsinchu 300091, Taiwan; meiyi@narlabs.org.tw (M.-Y.L.); pfhaung@narlabs.org.tw (C.-M.H.)
* Correspondence: yslai@narlabs.org.tw (Y.-S.L.); fhko@mail.nctu.edu.tw (F.-H.K.); Tel.: +886-03-577-3693-7532 (Y.-S.L.); +886-03-571-2121-55803 (F.-H.K.)

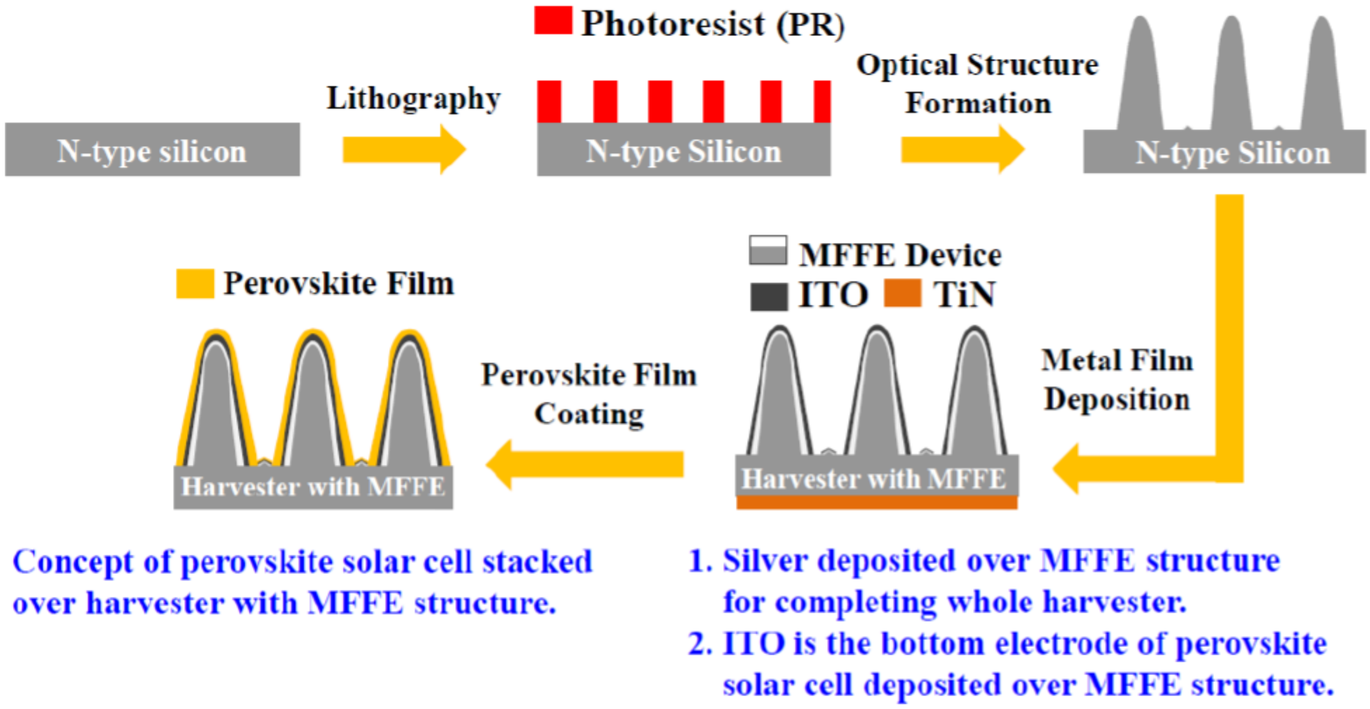

Figure S1. The brief flow for illustrating the design concept in this work about integrating solar harvester with the perovskite solar cell.
